# 

**Published:** 1888-06

**Authors:** 


CONTENTS:

ARTICLE I.—Implantation, with Clinical Demonstration. By M. H. Fletcher, D. D. S, M. D .... .... .... 49

" II.—A Case in Office Practice. By J. W. Jay, M. D., D. D. S. 55

" III.—Some Practical Points in the Physiology and Pathology of the Fifth Pair of Nerves in Relation to Invet erate Neuralgia. By Dr. Stretch Dowse ......... . 59

" IV.—Diseases Outside the Mouth that Affect the Oral Structures. By Dr. J. Y. Martin ..... ......... .... 64

" V.—Deciduous Teeth and the Evils of their Decay and Loss. By Dr. Garrett Newkirk ...... ..... . ..... . ..... .... 71

" VI.—The Infant-Food Problem. By Wm. B. Atkinson, M. D. 74

" VII.—A Case of Epithelioma of the Gums, with a Few Remarks. By Frank Lankester .... ....... ... ........... 80

" VIII.—Galvanoplastic Tooth Crowns. By Dr C. E. Diehl...... 84

" IX.—Cleft Palate and Artificial Velum. By Mr. J. A. Biggs . 89

EDITORIAL.

The Requisite Conditions for Practicing Dentistry in the Province of Quebec...... ...... ......................... ... ..... 93

